# Implications of prognosis-associated genes in pancreatic tumor metastasis: lessons from global studies in bioinformatics

**DOI:** 10.1007/s10555-021-09991-1

**Published:** 2021-09-30

**Authors:** Sophia G. Kisling, Gopalakrishnan Natarajan, Ramesh Pothuraju, Ashu Shah, Surinder K. Batra, Sukhwinder Kaur

**Affiliations:** 1 Department of Biochemistry and Molecular Biology, University of Nebraska Medical Center, Omaha, NE 68198-5870 USA; 2 Eppley Institute for Research in Cancer and Allied Diseases, University of Nebraska Medical Center, Omaha, NE USA; 3 Fred and Pamela Buffet Cancer Center, University of Nebraska Medical Center, Omaha, NE USA

**Keywords:** Molecular markers, Prognosis, Metastasis, Survival

## Abstract

Pancreatic cancer (PC) is a highly lethal malignancy with a 5-year survival rate of 10%. The occurrence of metastasis, among other hallmarks, is the main contributor to its poor prognosis. Consequently, the elucidation of metastatic genes involved in the aggressive nature of the disease and its poor prognosis will result in the development of new treatment modalities for improved management of PC. There is a deep interest in understanding underlying disease pathology, identifying key prognostic genes, and genes associated with metastasis. Computational approaches, which have become increasingly relevant over the last decade, are commonly used to explore such interests. This review aims to address global studies that have employed global approaches to identify prognostic and metastatic genes, while highlighting their methods and limitations. A panel of 48 prognostic genes were identified across these studies, but only five, including ANLN, ARNTL2, PLAU, TOP2A, and VCAN, were validated in multiple studies and associated with metastasis. Their association with metastasis has been further explored here, and the implications of these genes in the metastatic cascade have been interpreted.

## Introduction

With an incidence rate that has increased approximately 1% each year for the past 20 years, PC is currently ranked the 11^th^ most common cancer worldwide [1]. This year alone, the numbers of diagnoses and deaths attributed to this disease in the USA are projected to exceed 60,000 and 48,000, respectively. In fact, due to the consistently increasing incidence rate of PC, it is expected to surpass breast cancer as the third leading cause of cancer deaths by 2025 [2]. Currently, the stage-wide 5-year survival rate for PC is 10%, which is one of the lowest among all major cancers, demonstrating the need for novel detection and treatment modalities [3].

While the causes of PC are not completely understood, genetic factors, such as DNA mutations, have emerged as key factors in PC etiology. Genetic alterations can be hereditary, leading to disorders such as Lynch syndrome and Peutz-Jeghers syndrome. Further, lifestyle or environment, such as smoking, could cause mutations leading to the development of various malignancies. Additionally, the presence of certain benign conditions increases the risk for the development of pancreatic cancer. Diabetes mellitus and pancreatitis are two diseases that are commonly seen in patients prior to the development of PC [1, 4]. Interestingly, late-onset diabetes mellitus is also observed in individuals who have previously been diagnosed with PC, indicating a bi-directional association between these diseases [5]. However, understanding beyond PC etiology is urgently needed to develop effective interventions for improved diagnosis and treatment.

Over the last decade, treatment strategies have been actively exploring pathways associated with chemoresistance, metastasis, hypoxia, and immunosuppression for PC targeting. Further efforts are being made to explore the underlying heterogeneity of pancreatic tumors and target pathways associated with distinct subtypes [6–8]. Each of these components contributes to the recalcitrant nature of all major cancers, including PC, and explored therapies have been designed to take advantage of cell surface markers, metabolism, stroma, the immune system, and important signaling pathways, among other factors. However, we have yet to develop effective therapies which target the major attributes, specifically metastasis, which are responsible for the aggressive nature of PC.

## Gaps in the understanding of pancreatic tumor metastasis

Tumor metastasis is one of the main characteristics attributed to poor prognosis in PC. Due to its asymptomatic nature, PC is commonly diagnosed at late stage, when the cancer is locally advanced or metastasized, and treatment options are limited. In these aggressive tumors, excessive desmoplasia is often observed, and the metastases are primarily found in the liver, lung, and peritoneum [9]. Unfortunately, metastatic cases account for approximately 50% of new PC diagnoses, and their 5-year survival rate is a dismal 3% [3, 10]. Despite these facts, there is not yet a standard targeted therapy for metastasis, partly due to our limited understanding of systemic progression models and the molecular events that underlie the metastatic cascade.

The metastatic cascade consists of several fundamental processes, including invasion of the basement membrane, intravasation, survival in the circulation, extravasation, and colonization at secondary sites [11]. As cells progress through each of these steps, they undergo selective pressures, ensuring that only the fittest cells colonize the secondary tumor sites [12]. It begins when epithelial cells undergo a series of mutations that give them the metastatic capacity to invade the basement membrane, move across the extracellular matrix (ECM), and intravasate into the bloodstream. Once in the bloodstream, these metastatic tumor cells, or circulating tumor cells (CTCs), must acclimatize to the new environment and evade immune surveillance [13–15]. CTCs that have survived in the bloodstream and reached a distant organ, then referred to as disseminated tumor cells (DTCs), arrest and receive cues from the distant organ for the formation of micrometastases [16, 17]. While only a fraction of these CTCs and DTCs survive the selection process to form distant secondary tumors, their speculated role in recurrent cancer and drug resistance further implicates them in disease lethality [18, 19]. Moreover, this tumor cell motility, and consequently their metastasis, is a consequence of the dysregulation of cell adhesion molecules. This is highlighted by the long-standing hallmark of cancer that loss of E-cadherin expression is correlated with the migratory and invasive potential of the tumor cells both under *in vitro* and *in vivo* conditions [20].

There are currently two models that are commonly used to describe the metastatic process: the linear progression model and the parallel progression model [21]. The linear progression model, which is more widely recognized, proposes that the dissemination of tumor cells and the development of metastases occurs during late-stage primary tumor progression. The seeding cells (clones) from a late-stage tumor will harbor advanced mutations and growth potential, thereby allowing minimal genetic deviations between the primary tumor and metastases [22, 23]. Alternatively, the parallel progression model proposes early dissemination of tumor cells during the first stages of tumor progression. These early-stage cells have had limited genetic progression, and so the clones from this population are less genetically advanced compared to their linear counterpart. Moreover, early dissemination allows the primary tumor and metastases to evolve separately from a less advanced stage, leading to a higher level of genetic divergence between them [22, 23]. Although no direct evidence exists in support of either model, sequencing data and animal models of various cancer metastases, namely those of the liver, lung, and peritoneum, have provided indirect corroboration for both models, indicating that metastasis may not only be a cancer-specific but also a case-specific phenomenon [23].

The primary driver gene mutations of PC, including KRAS, TP53, p16/CDKN2A, and SMAD4, have a crucial role in early pancreatic lesions, local and advanced tumors, metastasis, and the hypovascular and hypoxic nature of the stroma, which contributes to the evasion of the immune response and alterations in cellular metabolism [24–26]. Tiwari et al. demonstrated that in PC, hypoxia-inducible factor 1 alpha (HIF1α) acts as a tumor suppressor by suppressing the expression of protein phosphatase 1 regulatory subunit 1B (PP1R1B), leading to the degradation of p53 protein in pancreatic cancer cells and an increase in the invasive and metastatic activity of tumors cells [27]. Another study found that methyltransferase-like 14 (METTL14) upregulation decreases p53 apoptosis effector related to PMP-22 (PERP) expression mediated through m^6^A modification and promotes pancreatic cancer metastasis [28]. Exosomes, which have become invaluable in cancer research, play an essential role in tumor initiation and the formation of extracellular signalosomes, which influence tumor microenvironment remodeling [29]. PC exosomes have also been shown to transport nucleic acids, proteins, or lipids from parental to recipient cells; produce pro-inflammatory cues; and facilitate immunosuppression, anti-apoptosis leading to angiogenesis, proliferation, and tumor metastasis. More specifically, exosomes produced by cancer-associated fibroblasts (CAFs), tumor-associated macrophages (TAMs), cancer-initiating cells (CICs), and pancreatic stem cells (PSCs) have a diverse potential of cellular functionalities such as growth, proliferation, drug resistance, epithelial-mesenchymal transition (EMT), migration, invasion, and metastasis [30]. Intriguingly, many investigators believe that pancreatic cancer metastasis is one of the primary causes of death. Yet, few studies envisage the molecular mechanism of the tumor cell journey to distant organs.

While many questions regarding the metastatic process remain elusive, our knowledge base thus far has demonstrated the importance of metastasis in disease lethality and the critical nature of developing targeted metastasis therapies. In other words, recent evidence indicates that metastasis is strongly associated with poor prognosis, and the targeting of these metastatic pathways may lead to improved patient outcomes [31]. Indeed, studies in breast, prostate, esophageal, and liver cancer have established a correlation between prognosis and the occurrence and progression of metastasis [32–38]. Specifically, the cell functions which are characteristic of metastasis, which include proliferation, angiogenesis, migration, and invasion, among others, correlate with prognostic gene signatures [32]. While there is no direct evidence of this relationship in PC, the aforementioned studies support the supposition that there may be an association between the stages of metastatic progression or the metastatic cascade and poor prognosis in PC. For these reasons, there has been extensive research on the mechanism of metastasis and prospective drug targets. Current targeted therapies, including the epidermal growth factor receptor (EGFR) inhibitor erlotinib and the neurotrophic tyrosine receptor kinase (NTRK)  inhibitors larotrectinib and entrectinib, are given to advanced-stage PC patients in combination with standard of care drugs to slow the progression of the tumor. This may stall local invasion, which occurs prior to the dissemination of cancer cells. However, these metastasis-inhibiting drugs are not always effective, and they do not serve in a neoadjuvant capacity [39].

Alongside our efforts to develop therapies that target the quintessential characteristics of these tumors, there has been an extensive search for prognostic markers, markers of metastasis, and associated biological pathways of PC. The expectation is that establishing a panel of prognostic genes for PC, specifically those associated with progression and metastasis, could lead to the identification of novel pathways for therapeutic targeting. In recent years, there has been a consensus that computational approaches in bioinformatics may be employed to better understand metastatic cascades and genes implicated in the aggressive nature of PC. In this review, we aim to address such studies and assess their findings while highlighting the merits and disadvantages of these approaches.

## Computational tools employed for identification of metastasis- and prognosis-associated genes

In recent years, computational approaches used to analyze gene data have become invaluable tools in cancer research. Identifying key genes involved in tumorigenesis, tumor pathogenesis, or the hallmarks of cancer, among other classifications, is crucial to our developing knowledge of the molecular mechanisms of cancer and our search for effective treatment targets. For particularly lethal cancers, such as PC, these computational methodologies are especially indispensable, as they may shed light on the genes which may be used for early diagnosis or play pivotal roles in the progression of the disease [40, 41].

There are numerous methods employed to determine differentially expressed genes between the phenotypes included in microarray datasets. The use of statistical software packages, such as Linear Models for Microarray Analysis (limma), GEO2R, and Weighted Gene Co-expression Network Analysis (WGCNA) in R programming language, is among the most common techniques used for this purpose. While identifying similar gene networks, limma determines the genes which can be attributed to each phenotype using a linear model, and WGCNA defines the differential co-expression networks between the phenotypes to identify differentially expressed genes (DEGs) [42]. GEO2R is a limma-based method also used for the analyses of differentially expressed pathways. These statistical packages are advantageous because they do not require prior command-line proficiency, making them a widely accessible resource.

Meta-analysis approaches utilized to analyze differentially expressed genes (DEGs), or subsets of genes identified from microarrays, are also highly diverse. A few of the most common resources include Gene Ontology (GO), Kyoto Encyclopedia of Genes and Genomes (KEGG), Gene Set Enrichment Analysis (GSEA), and the Database for Annotation, Visualization and Integrated Discovery (DAVID). These knowledge bases contain integrated analysis methods which utilize pathway databases such as KEGG, Reactome, BioCarta, and PANTHER to perform functional enrichment analysis. This identifies which biological processes, signaling pathways, or molecular functions are enriched in the genes of interest and demonstrates how they affect other genes and pathways to infer downstream effects. However, the best method for pathway analysis is highly dependent on the needs of the study and the statistical sensitivity required. For example, while DAVID is a highly efficient and comprehensive tool for gene set analysis, it is prone to false positives in pathway analysis. Alternatively, GSEA is arguably the least biased method for determining which pathways most significantly encompass the input genes, though it may also have limited sensitivity in gene set analysis [43]. Another interesting aspect of pathway analysis is that most methods integrate multiple resources and databases in order to have the most inclusive analysis; only a select few, such as KEGG pathway analysis and GSEA, have a single primary data source.

Protein–protein interaction networks and hub genes are also commonly explored in studies that utilize bioinformatics. Tools such as Cytoscape, Metascape, cBioPortal, and the Search Tool for the Retrieval of Interacting Genes/Proteins (STRING) allow for the analysis of genomics data, visualization of interactome networks of input genes, and identification of highly interconnected hub genes across given gene sets. Multiple databases comprising network and annotation data are integrated to support these tools, which helps elucidate genes or biological processes that may play a vital role in the phenotype of interest in a given dataset. Unlike the other tools, however, the STRING database incorporates both known and predicted interactions, and the type of interaction between genes is annotated in the analysis.

## Prognostic and metastatic markers as identified by computational approaches

The poor prognosis of PC is often associated with the presence of metastases in the lymph nodes and distant secondary sites. With that in mind, it is unsurprising that a number of the prognostic genes recognized by recent studies have been associated with the stages of the metastatic cascade in PC and other cancer types. Specifically, our review of all the literature has led to the collection of 48 prognostic genes, many of which are associated with various aspects of cancer progression. Among these genes, a set of five genes were differentially altered across multiple studies and implicated in the metastatic progression of PC. These include anillin actin-binding protein (ANLN), DNA topoisomerase II alpha (TOP2A), urokinase-type plasminogen activator (PLAU), versican (VCAN), and aryl hydrocarbon receptor nuclear translocator-like 2 (ARNTL2).

Nearly a dozen studies in the last 5 years have utilized bioinformatics analyses to identify genes associated with PC prognosis [44–54]. A recent study conducted by Luo et al. aimed to determine the molecular signatures for PDAC progression and a survival score to predict PDAC prognosis. Using PDAC data retrieved from GEO datasets GSE28735, GSE62452, and GSE57495, and DEGs, key genes associated with PDAC tumors, their association with prognosis, and their clinical significance were determined by assessment of WGCNA and miRNA profile survival analysis in R programming language. Kaplan–Meier survival analysis of metastasis data obtained from the International Cancer Genome Consortium (ICGC) demonstrated an association between several DEGs and metastasis. Further GO enrichment analysis, KEGG pathway analysis, and GSEA presented the biological processes, molecular functions, cellular components, and pathways that are enriched in these DEGs. Several genes were associated with poor prognosis, leading to a 7-gene signature which could accurately predict PDAC prognosis and metastasis: ARNTL2, desmoglein 3 (DSG3), protein tyrosine phosphatase receptor type R (PTPRR), ANLN, S100 calcium-binding protein A14 (S100A14), ankyrin repeat domain 22 (ANKRD22), and tetraspanin 7 (TSPAN7) [44]. The detailed annotation of these genes in PC progression and metastasis is included in Table 1).

**Table 1:** Recent studies that utilized computational methods to identify key genes in PC

Author	Year	PDAC datasets	Prognostic gene set identified	Validation method	Findings
Luo et al [44]	2021	GSE28735, GSE62452, GSE57495	ARNTL2, DSG3, PTPRR, ANLN, S100A14, ANKRD22, TSPAN7	Western blot (WB), RT-qPCR, IHC	Proposed gene panel is associated with poor survival, and predicts prognosis and metastasis
Xu et al [46]	2020	GSE19279, GSE42952, GSE71729	SCG5, CRYBA2, CPE, CHGB	qPCR	109 genes differentially expressed in metastatic PC; low expression of 4-gene prognostic panel of DEGs in PC indicates unfavorable prognosis
Jin et al [45]	2020	GSE32676, GSE15471, GSE71989	ANLN, ASPM, CDK1, CEP55, DTL, ECT2, NEK2, TOP2A, PRC1	WB	Discovered gene panel is associated with poor survival; CDK1 and CEP55 are positively associated with tumor grade
Lu et al [50]	2019	GSE15471, GSE19650, GSE32676, GSE71989	MET, MELK, SDC1, THBS1, TOP2A	In silico (TCGA)	5 hub genes identified as potential therapeutic targets; upregulated hub genes are significantly associated with prognosis
Zhou et al [49]	2019	GSE41368, GSE43795, GSE55643, GSE41369	MYC, SLC2A1, PKM, PLAU, PPARG, MET, ITGA3	Human Protein Atlas (HPA) IHC	7 hub genes and 10 miRNA targets predict survival; hub genes may have therapeutic potential
Ma et al [52]	2019	GSE15471, GSE16515, GSE41368, GSE62165, GSE62452, GSE71729, GSE71989, GSE91035	LAMC2, LAMB3, SERPINB5, AREG, SFRP4	In silico (TCGA)	10 hub genes associated with pathogenesis and five prognostic genes identified
Wu et al [48]	2019	GSE62165	PLAU, COL17A1	In silico (TCGA)	18 core hub genes found; 2 genes are significantly associated with poor prognosis
Wu et al [51]	2019	GSE71729, GSE62165, GSE62452, GSE28735, GSE15471, GSE16515, GSE32676	MET, KLK10, COL17A1, CEP55, ANKRD22, ITGB6, ARNTL2, MCOLN3, SLC25A45	In silico (GEO)	Established a 9 gene prognostic signature; upregulated genes were associated with poor survival
Chen et al [47]	2019	GSE15471, GSE16515, GSE6245	TOP2A, POSTN, PLAU, VCAN	NA	24 main hub genes were identified; 4 genes are associated with survival; VCAN associated with chemosensitivity
Li et al [53]	2019	GSE28735	SLC6A14, GALNT5, TSPAN1, IAPP	GEPIA (TCGA), qPCR	20 key hub genes were identified; four genes were associated with tumor stage; three genes were associated with poor prognosis
Lu et al [54]	2018	GSE62452, GSE15471, GSE102238, GSE16515, GSE62165	ITGA2, MMP7, ITGB4, ITGA3, VCAN, PLAU	In silico (GEO)	21 core hub genes identified; 6 genes proposed to predict poor survival

Anillin actin-binding protein (ANLN), ankyrin repeat domain 22 (ANKRD22), aryl hydrocarbon receptor nuclear translocator-like 2 (ARNTL2), proto-oncogene c-Met (MET), DNA topoisomerase II alpha (TOP2A), urokinase-type plasminogen activator (PLAU), and versican (VCAN) were included in the prognostic gene set across multiple studies and have been validated in both *in vitro* and in silico studies

Intriguingly, ANLN has been shown to play a role in the promotion of EMT in lung adenocarcinoma and cell–cell adhesion, migration, and invasion in PC [55, 56], indicating a potential role of ANLN in various stages of the metastatic cascade, including invasion of the basement membrane, intravasation, extravasation, and colonization at secondary sites (Table 2). While EMT is commonly recognized as a central aspect of cancer metastasis, it is the loss, rather than promotion, of cell adhesions that are associated with cancer development. However, the collective migration of cancer cells, a process where two or more cells whose cell–cell junctions are intact move together into nearby tissues and vasculature, could be one explanation for ANLN-mediated cell–cell adhesion [57, 58]. As this type of migration would require remodeling of the extracellular matrix (ECM) to accommodate the movement of these cell groups, and these changes would incite cell motility through integrins, the enriched pathways support this supposition for ECM disassembly, ECM organization, collagen catabolic process, integrin binding, and cell migration, which have been observed in PC DEGs.

**Table 2:** Genes associated with the stages of the metastatic cascade

Gene	Function	Protein expression and localization in PC		Association with cellular movement	Potential involvement in the metastatic cascade	Associated cancers	Associated PC progression pathways	Ref
		Expression	Cellular location					
Anillin actin-binding protein (ANLN)	Contributes to cytoskeleton integrity and cleavage furrow formation	+	Cytoskeleton, nucleus	EMT, cell–cell adhesion, migration, invasion, proliferation, metastasis	Invasion of the basement membrane, intravasation, extravasation to secondary sites, colonization at secondary sites	Bladder cancer, lung cancer, pancreatic cancer	EZH2/miR-218-5p/LASP1 signaling axis, HMGA2/ANLN signaling axis, p53 signaling pathway	[56, 59–62]
DNA topoisomerase II alpha (TOP2A)	Binds to DNA and changes strand topology	+	Cytoplasm, nucleus	EMT, migration, invasion, proliferation, metastasis	Invasion of the basement membrane, intravasation, survival in the circulation, extravasation to secondary sites, colonization at secondary sites	Bladder cancer, cervical cancer, lung cancer, pancreatic cancer	miR-139/TOP2A/β-catenin axis, p53 signaling pathway	[63–67]
Urokinase-type plasminogen activator (PLAU)	Cleaves plasminogen to form active plasmin	+	Secreted	EMT, invasion, migration, metastasis	Invasion of the basement membrane, intravasation, extravasation to secondary sites, colonization at secondary sites	Breast cancer, fibrosarcoma, pancreatic cancer, prostate cancer	uPAR/ERK/p38 signaling pathway, Rel/NF-κB signaling pathway	[68–74]
Versican (VCAN)	Proteoglycan, cell adhesion to the ECM	+	ECM, secreted	Invasion, metastasis, ECM remodeling, migration, inhibits cell adhesion to ECM	Invasion of the basement membrane, intravasation, extravasation to secondary sites, colonization at secondary sites	Breast cancer, colorectal cancer, prostate cancer, kidney cancer, melanoma, pancreatic cancer	Post-translational modifications of VCAN (specified pathways not available)	[75–80]
Aryl hydrocarbon receptor nuclear translocator-like 2 (ARNTL2)	Activates transcription of circadian rhythm genes	+	Nucleus	Invasion, migration, proliferation, metastasis	Invasion of the basement membrane, intravasation, survival in the circulation, extravasation to secondary sites, colonization at secondary sites	Colon cancer, colorectal cancer, lung cancer, pancreatic cancer	TFGβ-FAK signaling	[81–84]

ANLN, TOP2A, PLAU, VCAN, and ARNTL2 may play a role in the initial and final stages of metastasis, where invasion and migration are key characteristics. TOP2A and ARNTL2  expression further impact the survival of circulating tumor cells (CTCs). + indicates the positive expression of the gene and proteins in pancreatic cancer

The metastatic significance of ARNTL2 expression has also been explored in lung adenocarcinoma and PC. In lung adenocarcinoma, ARNTL2 expression is an important factor in the survival of DTCs and CTCs and metastatic seeding [81]. In a similar manner, its expression in PC has been shown to positively regulate the TGF-β signaling pathway, which has been observed to promote metastasis when expressed in tumors and is known to promote tumor progression in PC [82, 85]. ANTL2 is also observed to promote cell focal adhesion in PC, which contributes to cell dissociation from the primary tumor and reattachment to the ECM for invasion and intravasation [82, 86]. These findings indicate that ARNTL2 may be expressed throughout the entire metastatic cascade, including invasion of the basement membrane, intravasation, survival in the circulation, extravasation to secondary sites, and colonization at secondary sites (Table 2). The enriched pathways for integrin binding, laminin-binding, ECM disassembly, and PI3K-AKT signaling pathway in PC further emphasize the metastatic involvement of ARNTL2, as they are associated with invasion and metastasis, and there is a link between TGF-β and PI3K-AKT signaling pathways, demonstrated by crosstalk in cancer [85, 87].

Studies have also aimed to delineate the genes associated with PC initiation, progression, and prognosis. Jin et al. designed a study to identify genes responsible for the molecular mechanism of PC tumorigenesis and proliferation. DEGs were determined from GEO datasets GSE32676, GSE15471, and GSE71989 using GEO2R. DAVID and the STRING database were used to classify the enriched biological processes, molecular functions, cellular components, and pathways, while the PPI network for the DEGs was built using Cytoscape. Hub genes and their mode of regulation were also identified using Cytoscape, and their association with poor survival was determined by constructing Kaplan–Meier curves via cBioPortal. Further, expression changes throughout disease progression were explored for each DEG using Oncomine, which demonstrated increased expression of cyclin-dependent kinase 1 (CDK1) and centrosomal protein 55 (CEP55) as PC progresses. They identified 10 hub genes that were associated with decreased survival: ANLN, assembly factor for spindle microtubules (ASPM), CDK1, CEP55, denticleless E3 ubiquitin protein ligase homolog (DTL), epithelial cell transforming 2 (ECT2), NIMA-related kinase 2 (NEK2), TOP2A, and protein regulator of cytokinesis 1 (PRC1) [45] (Table 1).

Like ANLN, TOP2A positively regulates invasion and migration and promotes EMT in lung adenocarcinoma, and in PC, TOP2A expression gives rise to enhanced cell proliferation, migration, and EMT [63, 64]. These downstream effects of TOP2A expression, along with the enhanced expression of TOP2A observed in metastatic luminal breast cancer and prostate cancer, indicate a potential role in all five stages of the metastatic cascade [88–91] (Table 2). This is further reflected in the upregulated pathways observed in PC DEGs, comprising integrin binding, laminin-binding, collagen catabolic process, and cell migration, as integrins are key molecules for migration, the proteolytic breakdown of collagen is important in an invasion, and laminin and collagen-binding are significant in intravasation and extravasation.

Xu et al. recently investigated potential prognostic genes and the molecular mechanisms of PC metastasis. A total of 109 DEGs in metastatic PC were determined from GEO datasets GSE19279, GSE42952, and GSE71729 using the limma package in the R programming language. Annotation of function, pathway analysis, PPI network analysis, and prognostic analysis of these genes were performed using DAVID, the STRING database, Cytoscape, and the GEPIA analysis tool. Decreased survival was significantly associated with the low expression of four DEGs, including secretogranin V (SCG5), crystallin beta A2 (CRYBA2), carboxypeptidase E (CPE), and chromogranin B (CHGB), indicating that the abnormal regulation of these genes in cancer may impact overall survival and the metastatic properties of PC tumors [46] (Table 1).

In recent years, seven studies explored potential gene targets of PC and genes associated with prognosis and progression of the disease using similar techniques. The interest of a study by Chen et al*.* lay in the use of integrated bioinformatics to identify genes involved in PC tumorigenesis. DEGs were screened from GEO datasets GSE15471, GSE16515, and GSE6245 using GEO2R. The functional significance, enriched pathways, and PPI network for the common DEGs from the three datasets were determined using DAVID, the STRING database, and Cytoscape. Cytoscape also allowed for the identification of 24 main hub genes, and their association with poor survival was determined through cBioPortal Kaplan–Meier curves. The authors identified four genes with differential survival: TOP2A, periostin (POSTN), PLAU, and VCAN (Table 1). The ROC curves of these genes identified a significant area under the curve (AUC), indicating that they may also have diagnostic potential. VCAN was determined as a relatively novel marker for PC progression and further exploration using Oncomine and the R2 Genomics Analysis and Visualization Platform, an online genomics analysis tool, suggested that VCAN expression may play an important role in PC response to chemotherapy treatment [47].

Overexpression of VCAN has been observed in numerous cancers and their respective metastases but has only been associated with the invasion and motility of breast cancer, prostate cancer, PC, and melanoma, and invasion and migration in renal cell carcinoma [75–79]. As a major component of the ECM, its influence on cell adhesion, cell migration, and cell invasion is logical. Further, as invasion is a key aspect of the metastatic process, it can be surmised from these findings that VCAN may be involved in the invasion of the basement membrane, intravasation, extravasation to secondary sites, and colonization at secondary sites, which are all stages of the metastatic cascade that depend on the invasive nature of cancer cells (Table 2).

It is plausible that PLAU may function as a promoter of metastasis at the beginning and end of the metastatic cascade. The primary function of its protein product, uPA is involved in remodeling ECM, which in breast and cervical cancer enhances cancer cell motility and ability to invade the basement membrane and migrate, thereby promoting metastasis through proteolytic destruction of ECM [92, 93]. Intravasation and extravasation are similarly associated with EMT and ECM degradation, while colonization at secondary sites is associated with the motility and invasion of cancer cells (Table 2). ECM disassembly and protein activation cascade, which were enriched pathways of PC DEGs, highlight the function of PLAU and its potential role in the metastatic cascade.

Wu et al. further explored genes that may be associated with PC prognosis. Unlike many other studies, only a single GEO dataset, GSE62165, was used to identify DEGs with the limma package in R programming language. While this limits the sample number, the use of one large size dataset is beneficial in that normalization is not needed, and the available clinical study data is uniform. Clusterprofiler in R programming language classified the enriched biological processes, molecular functions, cellular components, and pathways for DEGs, and their PPI network was constructed using the STRING database. Cytoscape and UALCAN, an online tool for analyzing omics data, were used to identify 18 core genes and their association with survival. They found two genes, PLAU and collagen type XVII alpha 1 chain (COL17A1), associated with poor prognosis [48] (Table 1).

In a similar manner, Zhou et al. investigated biomarkers and prognostic targets for PDAC. As microRNAs (miRNAs) have been previously shown to have diagnostic and prognostic potential in PC, DEGs and differentially expressed miRNAs were determined from GEO datasets GSE41368, GSE43795, GSE55643, and GSE41369, which were analyzed using GEO2R. Commonly identified DEGs from all four GEO datasets were further analyzed using Metascape, GO analysis, KEGG pathway analysis, the STRING database, Cytoscape, and FunRich, a tool for analyzing omics data, for functional enrichment. The functional analysis of these DEGs helped determine their associated biological processes, molecular functions, cellular components, enriched pathways, and PPI networks. Hub genes were identified using Cytoscape, and survival analysis of these genes was performed using the KM plotter. miRNAs were similarly analyzed, and the overlap between the miRNA gene targets and DEGs revealed seven hub genes, including proto-oncogene c-Myc (MYC), solute carrier family 2 member 1 (SLC2A1), pyruvate kinase M1/2 (PKM), PLAU, peroxisome proliferator-activated receptor gamma (PPARG), proto-oncogene c-Met (MET), and integrin subunit alpha 3 (ITGA3), which may be associated with poor prognosis, and whose miRNA regulators are associated with EMT and the PI3K-AKT and MAPK/ERK signaling pathways, among other metastatic processes [49] (Table 1).

In a study by Lu et al., hub genes that could be used as targets in PC diagnosis and treatment strategies were elucidated. The limma package in R programming language was used to determine DEGs from GEO datasets GSE15471, GSE19650, GSE32676, and GSE71989. DAVID, KEGG Orthology Based Annotation System (KOBAS) enrichment, the STRING database, and Cytoscape were used to classify the enriched biological processes, molecular functions, cellular components, and pathways for the DEGs, as well as their PPI network. Hub genes and their association with survival were identified and explored using Cytoscape and UALCAN analysis. Five hub genes were found to be associated with decreased survival: MET, maternal embryonic leucine zipper kinase (MELK), syndecan 1 (SDC1), thrombospondin 1 (THBS1), and TOP2A [50] (Table 1).

Unlike prior studies, Wu et al. developed a prognostic signature and nomogram, a statistical model used for risk prediction, which could be used to predict overall survival in PC. The limma package in R programming language was used to analyze GEO datasets GSE71729, GSE62165, GSE62452, GSE28735, GSE15471, GSE16515, and GSE32676 to identify DEGs. They further explored enriched biological processes through GO enrichment and KEGG pathway analysis. Signaling pathways and the PPI network of the DEGs were identified with DAVID and the STRING database. Cytoscape was used to identify hub genes of the PPI network, and their prognostic potential was explored and tested through Cox regression analysis, Kaplan–Meier analysis, ROC curves, and Harrell’s concordance index. Nine prognostic genes, including MET, kallikrein-related peptidase 10 (KLK10), COL17A1, CEP55, ANKRD22, integrin subunit beta 6 (ITGB6), ARNTL2, mucolipin TRP cation channel 3 (MCOLN3), and solute carrier family 25 member 45 (SLC25A45), were identified from these analyses [51] (Table 1).

Genes associated with the pathogenesis and tumorigenesis of PDAC were identified through integrative meta-analysis by Ma et al. DEGs were determined from GEO datasets GSE15471, GSE16515, GSE41368, GSE62165, GSE62452, GSE71729, GSE71989, and GSE91035 using the limma package in R programming language. Common DEGs were identified through robust rank aggregation in R programming language, which confirmed only the most statistically significant genes. The functional significance, pathway enrichment, PPI network, and survival association for the common DEGs were explored using GO enrichment analysis, KEGG pathway analysis, the STRING database, Cytoscape, Cox regression analysis, and Kaplan–Meier analysis. Ten genes were associated with pathogenesis, comprising of albumin (ALB), epidermal growth factor (EGF), matrix metallopeptidase 9 (MMP9), epidermal growth factor receptor (EGFR), fibronectin 1 (FN1), matrix metallopeptidase 1 (MMP1), serpin family E member 1 (SERPINE1), TIMP metallopeptidase inhibitor 1 (TIMP1), PLAU, and urokinase-type plasminogen activator receptor (PLAUR). Laminin subunit gamma 2 (LAMC2), laminin subunit beta 3 (LAMB3), serpin family B member 5 (SERPINB5), amphiregulin (AREG), and secreted frizzled-related protein 4 (SFRP4) were reported to associate with PDAC prognosis and potential as a prognostic signature [52] (Table 1).

Li et al. used computational approaches to identify therapeutic targets for PC and gain insight into the underlying molecular mechanisms of PDAC using a bioinformatics approach. To determine DEGs, GEO dataset GSE28735 was analyzed using GEO2R. The biological processes, molecular functions, cellular components, and pathway enrichment were classified using DAVID. The mRNA expression of the top DEGs and their association with survival were determined using boxplot analysis and survival analysis from the GEPIA web tool, and identified 20 key hub genes from the PPI network of DEGs using the STRING database. Four genes were associated with the tumor stage, including solute carrier family 6 member 14 (SLC6A14), polypeptide N-acetylgalactosaminyltransferase 5 (GALNT5), tetraspanin 1 (TSPAN1), and islet amyloid polypeptide (IAPP). Excluding IAPP, which was associated with a favorable prognosis, these genes were also associated with poor prognosis [53] (Table 1).

Novel hub genes and pathways which can be utilized to diagnose, predict the prognosis of, or treat PDAC were recently explored in a study by Lu et al. For determining hub genes, GEO datasets GSE62452, GSE15471, GSE102238, GSE16515, and GSE62165 were analyzed using GEO2R. A total of 21 core upregulated hub genes were consistently present in all five GEO datasets. They were further explored using DAVID, the STRING database, Cytoscape, and OncoLnc, a tool for analyzing survival data and correlated RNA expression, to elucidate their biological function, enriched pathways, PPI network, and survival association. Six core hub genes were associated with decreased survival and may be potential clinical markers: integrin subunit alpha 2 (ITGA2), matrix metallopeptidase 7 (MMP7), integrin subunit beta 4 (ITGB4), ITGA3, VCAN, and PLAU [54] (Table 1).

**Fig. 1:**
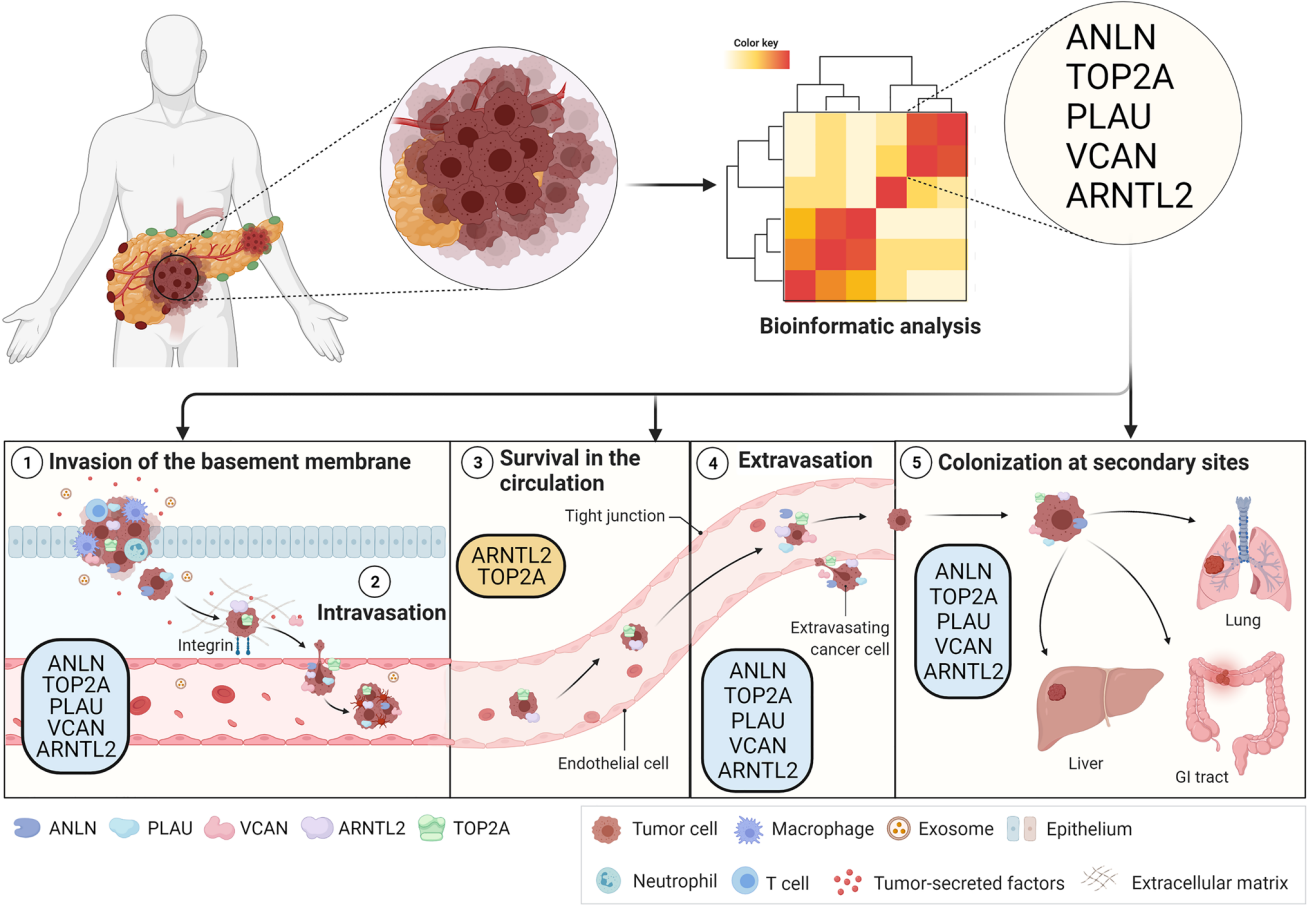
Schematic of metastatic cascade in PC. Multiple in silico studies have identified prognostic and metastatic gene sets in PC. By reviewing all the literature, we found a total of 48 prognostic genes, and further identified a set of five genes which were observed in multiple studies and found to be implicated in the metastatic progression of PC. These includes anillin actin-binding protein (ANLN), DNA topoisomerase II alpha (TOP2A), urokinase-type plasminogen activator (PLAU), versican (VCAN), and aryl hydrocarbon receptor nuclear translocator-like 2 (ARNTL2). The invasion, metastasis, and migration of tumor cells are common characteristics influenced by these genes, implicating them in the early and late stages of the metastatic cascade. ANLN is exclusively associated with the collective migration of tumor cells to secondary sites through the promotion of cell–cell adhesions, and ANTL2 and TOP2A are uniquely associated with tumor cell survival while in circulation

**Fig. 2:**
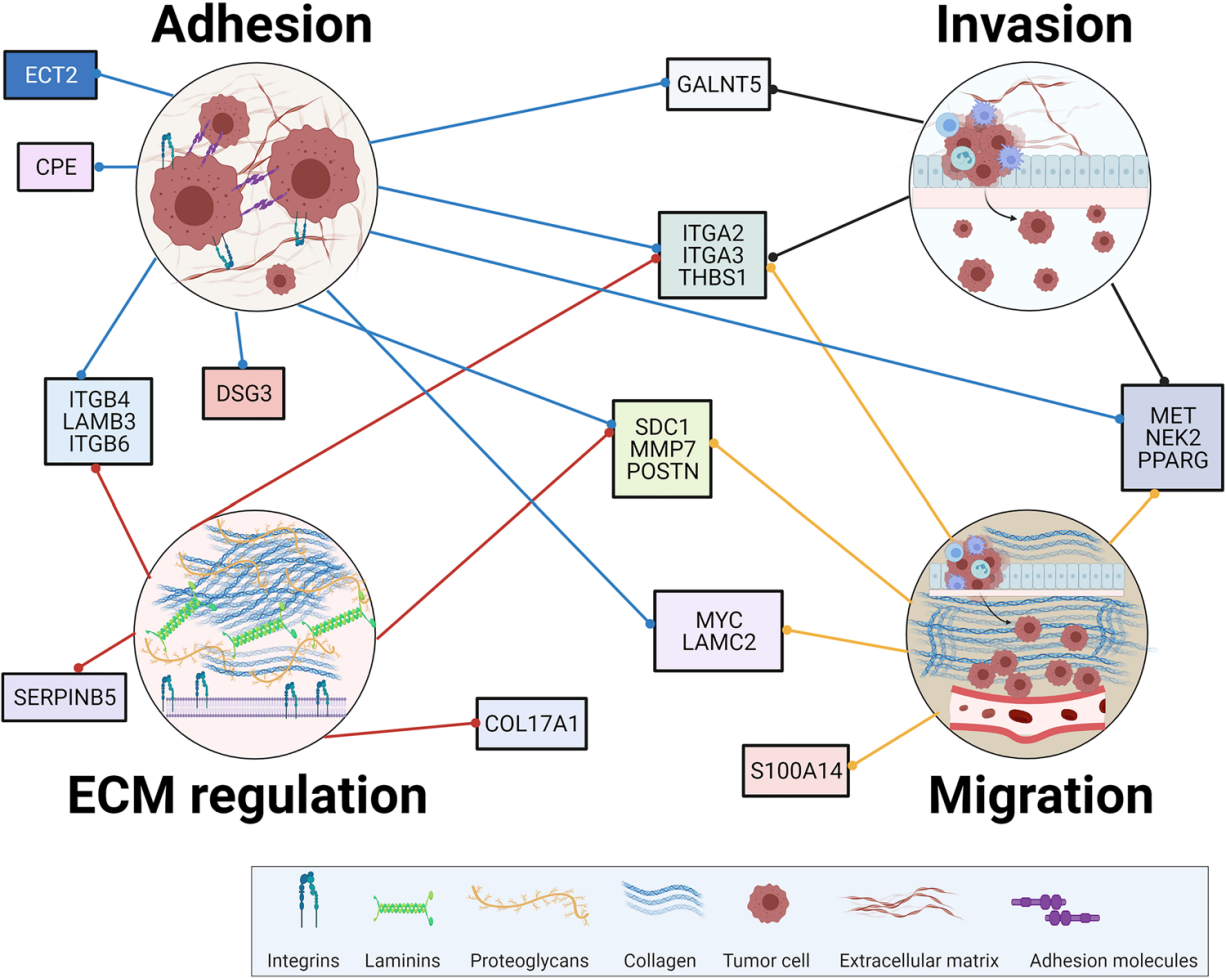
Meta-analysis of prognostic genes identified across multiple studies. Among the 48 computationally-derived prognostic genes, 21 were found to play a role in processes involved in metastasis, including adhesion, invasion, ECM regulation, and migration in PC. Interestingly, several of these genes, including integrin subunit alpha 2 (ITGA2), integrin subunit alpha 3 (ITGA3), thrombospondin 1 (THBS1), syndecan 1 (SDC1), matrix metallopeptidase 7 (MMP7), periostin (POSTN), proto-oncogene c-Met (MET), versican (VCAN), NIMA related kinase 2 (NEK2), and peroxisome proliferator activated receptor gamma (PPARG), were observed to play a role in a number of these processes, indicating that they may also be important in the metastatic cascade

In totality, 48 prognostic genes were identified, including ANKRD22, ANLN, AREG, ARNTL2, ASPM, CDK1, CEP55, CHGB, COL17A1, CPE, CRYBA2, DSG3, DTL, ECT2, GALNT5, IAPP, ITGA2, ITGA3, ITGB4, ITGB6, KLK10, LAMB3, LAMC2, MCOLN3, MELK, MET, MMP7, MYC, NEK2, PKM, PLAU, POSTN, PPARG, PRC1, PTPRR, S100A14, SCG5, SDC1, SERPINB5, SFRP4, SLC25A45, SLC2A1, SLC6A14, THBS1, TOP2A, TSPAN1, TSPAN7, and VCAN. Several studies identified ANLN, ANKRD22, ARNTL2, MET, TOP2A, PLAU, and VCANas prognostic genes in PC, and of those, ANLN, ARNTL2, TOP2A, PLAU, and VCAN have also been implicated in metastasis. Their impact on several key metastatic processes, including EMT, proliferation, adhesion, invasion, and migration, has been demonstrated in multiple cancer types [55, 56, 63, 64, 75–79, 82, 85, 86, 88–93]. From these studies, we can infer the stages of the metastatic cascade which are impacted by the regulation of these genes. Specifically, ANLN, ARNTL2, TOP2A, PLAU, and VCAN expression are important in the invasion of the basement membrane, intravasation, extravasation, and colonization at secondary sites, but only TOP2A and ARNTL2 contribute to the survival of cancer cells while in circulation (Fig. 1). Moreover, while not directly connected with metastasis, ECT2, CPE, DSG3, ITGB4, LAMB3, ITGB6, SERPINB5, COL17A1, GALNT5, ITGA2, ITGA3, THBS1, SDC1, MMP7, POSTN, MYC, LAMC2, S100A14, MET, NEK2, and PPARG have been implicated in processes associated with the metastatic cascade [44–54] (Fig. 2).

## Significant pathways in PC associated with newly identified prognostic signature and metastasis

Several significantly enriched pathways were identified among all the studies aimed to identify prognostic, therapeutic, or metastatic genes. For the studies which performed GO enrichment analysis for combined upregulated and downregulated DEGs, enrichment was commonly depicted in the biological processes and molecular functions associated with ECM maintenance and cell adhesion and migration [47, 49–51]. Interestingly, one study performed clustering analysis on DEGs prior to enrichment analysis, highlighting additional biological pathways, including blood vessel development, vasculature development, smooth muscle development, and cell junction assembly [51]. Likewise, the studies with combined DEGs had similar terms returned from KEGG pathway analysis, which described the most significant enrichment in ECM-receptor interaction, focal adhesion, and the PI3K-AKT signaling pathway [47, 49–51].

Receptor tyrosine kinases (RTKs) are membrane-bound receptors that initiate signaling events upon binding to growth factors, hormones, cytokines, neurotrophic factors, and, pertinently, extracellular signaling molecules and ECM components. RTKs act in conserved pathways that involve signaling events of cellular proliferation, differentiation, survival, and migration in cancer [94]. RTK signaling is tightly regulated under normal conditions but can be aberrantly dysregulated upon oncogenic insults. For example, the enhanced signaling of epidermal growth factor receptor (EGFR), one of the members of the ERBB family of RTKs comprising ERBB1 (EGFR), ERBB2, ERBB3, and ERBB4, is implicated in the development of many solid tumors [94]. RTKs exert their signaling by auto- and transphosphorylation of their intracellular C-terminal region. This can activate many important signaling pathways, including PI3K-AKT, RAS/RAF/MAPK, JAK-STAT, and PLC-γ1, which govern cancer cell events and functionalities such as proliferation, metabolism, angiogenesis, progression, and survival [95–97].

Numerous studies have elucidated the role of EGFR in the early and late stages of pancreatic cancer progression. Though KRAS activation is a prerequisite for the initial stages of the progression and is found to be mutated in 90% of PC patients, EGFR activity is also important for inducing PC progression through MEK/ERK activity [98]. The challenges in combating PC are majorly due to recurrence, metastatic events, and drug resistance attributed to cancer stem cell populations residing in the tumor bulk. It was recently shown that afatinib, a pan-EGFR inhibitor, reduces SOX9, a key molecule in maintaining stem cell populations. As the EGFR/ERK/FOXA2/SOX9 axis regulates pancreatic cancer stem cells (PCSCs), inhibition of EGFR hampers the growth and motility of PCSCs mediated through this axis [99]. Provided that EGFR signaling activates many pathways associated with carcinogenesis and metastasis, such as MAPK, PI3K, and JAK-STAT, it is no surprise that it has made an attractive target for cancer therapy. Various studies have described small molecule EGFR inhibitors, including erlotinib, gefitinib, afatinib, and osimertinib (AZD9291) [100, 101]. In addition, specific monoclonal antibodies against EGFR such as cetuximab and panitumumab are FDA approved for various cancer indications [102, 103]. Among small molecule inhibitors, erlotinib is FDA approved and is used in clinics in combination with gemcitabine for treating PC patients with local, advanced, unresectable, or metastatic tumors [104].

The consensus of GO enrichment analysis terms for upregulated DEGs among studies that performed separate analyses for each set of DEGs included pathways for biological process concerning ECM maintenance, collagen catabolic process, cell migration, and cell adhesion. Similarly, enrichment in the molecular function category for upregulated DEGs included ECM structure, collagen binding, integrin binding, cell adhesion molecule binding, and cadherin binding involved in cell–cell adhesion. In support of this, cellular component enrichment for upregulated DEGs was found in the ECM and cell junctions [44–46, 48, 52–54]. Further, these features play key roles in migration and EMT, which are predominantly associated with metastasis.

PC metastasis is mediated through a culmination of environmentally derived cell-intrinsic and cell-extrinsic cues, which give tumor cells the ability to migrate from the primary tumor to distant organs. Extrinsic cues, including paracrine and autocrine mechanisms, enable these tumor cells to reach the destined organ for colonization [105]. Extrinsic cues can also cause phenotypic changes in neoplastic cancer cells, which must be attained in order to gain motility and the ability to cross the hurdles of physical restrictions and vasculature. EMT is the trans-differentiation cellular process where epithelial cells acquire a mesenchymal phenotype via a series of biochemical changes induced by several growth factors, including TGF-β, HGF, EGF, IGF, and FGF [106, 107]. In the prerequisite phenotype, cells lose their epithelial characteristics (markers like E-cadherin, occludin, claudin, and laminin-1) and switch to mesenchymal traits (N-cadherin, vimentin, and fibronectin), causing the dysregulation of cell–cell contacts and the dissociation of cells from the epithelial layer [108]. Many transcriptional regulators can regulate this mesenchymal phenotype. TWIST, SNAIL1, SNAIL2, ZEB1, and ZEB2 repress E-cadherin expression and activate mesenchymal differentiation markers such as N-cadherin and vimentin, cellular matrix and focal adhesion proteins, and matrix metalloproteinases involved in promoting motility [109–111]. Additionally, calreticulin, a calcium-binding endoplasmic reticulum protein known to have various cellular roles, including that of a chaperone, has been shown to promote EMT through the Integrin/EGFR-ERK/MAPK axis in PC [112–114]. Longping Go-Ichi-Ni-San 2, an oncogene, is known to have a role in clinically advanced stages of PC through activation of the ERK/MAPK signaling pathway, which might mediate EMT. The reverse process of EMT, a concept called mesenchymal to epithelial transition (MET), occurs once metastatic tumor cells reach distant organs and colonize, and utilize many of the same pathways [24, 115–117]. This stresses the importance of genes and pathways involved in the process of EMT and points to their potential as therapeutic targets for metastasis.

Integrins belong to the cell adhesions family and comprise 24 αβ heterodimers formed from different α and β subunits. Integrin-β1, for example, associates with multiple α subunits to create 12 receptors for ECM components like collagen, laminin, and fibronectin rich in arginine-glycine-aspartic acid [118, 119]. They are the integral receptors that mediate cell adhesion and function as a mechanotransmitter for oncogenic and metastatic signals. Their interaction with the ECM involves the organization of the cytoskeleton and relaying intracellular signals from the ECM to regulate survival, proliferation, migration, and EMT, among other cell fate transitions [120, 121]. Integrin-facilitated cell adhesion to the extracellular matrix is highly controlled and, upon dysregulation, causes pathogenesis. In the case of pancreatic cancer, this elicits phenotypes and signaling pathways conducive to tumor growth and migration [122]. Further, it has been shown that integrin-β1 is involved in ERK signaling, and its inhibition decreases KRAS signaling in PC cells lacking ECM attachment [123, 124]. Integrin-β1 has also been implicated in the activation of PI3K signaling in PC [122]. The significance of integrins in cancer is further implicated by integrin-β8, which plays a vital role in PC cell radiochemoresistance, intracellular vesicle trafficking, and autophagy upon irradiation [125].

For downregulated DEGs, GO pathway enrichment for the biological process category included those related to digestion and proteolysis, organismal homeostasis, collagen maintenance, and ECM maintenance. The molecular function category for downregulated DEGs mainly encompassed peptidase and lipase activity, and cell adhesion. Enrichment of downregulated DEGs in the cellular component category was found in the ECM, vesicles, endoplasmic reticulum lumen, and, uniquely, platelet alpha granules [44, 46, 48, 52–54].

KEGG pathway analysis of upregulated and downregulated DEGs revealed differential enrichment of upregulated DEGs in the interleukin-17 signaling pathway and the PPAR signaling pathway. Pathways differentially enriched in downregulated DEGs, as determined by KEGG pathway analysis, included pancreatic secretion, and complement and coagulation cascade pathways [44–46, 48, 52–54]. The most significantly enriched pathway for upregulated DEGs, and most common across studies, was the PI3K-AKT signaling pathway.

In PC, nearly 59% of patients have elevated PI3K-AKT signaling. This pathway is normally regulated by phosphatase and tensin homolog (PTEN), a natural antagonist of PI3K, but this tumor suppressor is often lost in cancer [126, 127]. Similarly, about 60% of PC patients have increased expression of AKT2, which acts as an oncogene that presides over many cellular processes, including survival. AKT2 is the major downstream effector for the PI3K and RTK pathways [126, 128, 129]. PI3K/AKT/mTOR elicits signaling events responsible for regulating many essential cellular processes, including cell growth, metabolism, survival, metastasis, and resistance to chemotherapy [130]. It has also been established that this pathway plays a vital role in angiogenesis, macrophage transcriptional reprogramming, T cell differentiation, tumor cell homeostasis, and fibroblast-supported chemoresistance, apoptosis, invasion, tumorigenesis, and EMT [128, 131–134]. Further, PI3K signaling in stromal cells modulates the surrounding microenvironment, creating a space conducive for metastatic events. Its dysregulation leads to oncogenic signals, which involve changes in proliferation, migration, and immune modulations [135–138]. Unsurprisingly, the frequent amplification, mutation, or loss of key PI3K/AKT/mTOR regulators in many solid cancers has made it an attractive therapeutic target [139, 140]. Everolimus, an mTOR inhibitor, has been studied in numerous PC clinical trials. However, poor efficacy has been observed for everolimus alone and in combination with various standard of care therapies. It is postulated that the identification of drugs that are synergistic with everolimus may result in more successful responses [141].

Remarkably, many of the enriched pathways for the upregulated genes were associated with invasion, metastasis, tumorigenesis, and angiogenesis. In contrast, enriched pathways for downregulated genes were concerned with cellular maintenance, homeostasis, and inhibition of tumor progression. Inflammation and immune response pathways, tumor cell adhesion and motility pathways, lipid uptake and processing pathways, and the PI3K-AKT signaling pathway were enriched for both upregulated and downregulated genes. The upregulation of genes involved in invasion, metastasis, and angiogenesis falls in line with the expected aggressive nature of PC. The presence of shared immune pathways, cell movement, and energy pathways between these upregulated and downregulated genes may point to the regulatory role of the tumor microenvironment or indicate that they are complexly regulated throughout pancreatic tumorigenesis and tumor progression.

## Similarities and limitations of study design

Due to the limited availability of publicly available microarray datasets, many studies utilized at least one common dataset to define a prognostic signature. The data availability is even less for the studies interested in metastatic genes. Interestingly, while the use of these datasets led to the identification of similar upregulated and downregulated pathways across studies, the pipeline employed by each group was unique, and therefore, very few genes overlapped across each group’s prognostic gene set. However, multiple members belonging to the same gene families were identified, and DEGs had a moderate degree of similarity between studies. It is important to note that despite the distinct pipeline of each study, the methodologies across them are comparable. While R programming language was commonly used to identify DEGs in many of these studies, DAVID, the STRING database, and Cytoscape were heavily relied on to discover the biological functions, enriched pathways, and the PPI network of DEGs. Remarkably, GSEA was used to identify the enriched pathways of DEGs in only one study, possibly due to the low sensitivity of this analysis.

Moreover, methods like STRING and GO enrichment analysis rely on experimental annotations and those derived electronically. While this increases the number of annotations available for analysis, it can lead to the assignment of incorrect ones, as electronic annotations are not as accurate as their experimental counterparts [142]. Unfortunately, there is currently no way to limit analyses to experimental annotations, and unlike STRING, GO enrichment analysis does not distinguish between the two in the output. Further verification of these annotations is often needed to determine their true functional significance.

The key difference among these studies is how DEGs were grouped for analysis. Four of these studies performed pathway analyses on DEGs as one group, while seven studies divided DEGs into upregulated and downregulated categories. Though a holistic look at the pathway enrichment of all DEGs can be insightful and elucidate which pathways are altered in pancreatic tumors as compared to the normal pancreas, the scope of this analysis is limited. When considering all DEGs, we are unable to determine whether increased or decreased expression is associated with the altered pathways. The separate analysis of upregulated and downregulated genes allows us to better understand whether the pathway changes are due to increased or decreased expression of specific genes. In other words, with differentiation of upregulated and downregulated DEGs, we can recognize with greater clarity the molecular basis of important pathway changes between normal pancreas and pancreatic tumors.

## Conclusion

The use of integrated bioinformatics has allowed us to identify key genes common in many malignancies. In PC, these methodologies have aided in determining genes associated with prognosis, tumorigenesis, and, as recently shown, metastasis. The knowledge acquired from these studies will help develop biomarkers and drugs specifically targeting these processes and lead to better disease management. Further, PC prognosis is often dependent on the presence of metastases. Metastasis is a multi-stage process; we must understand which stage has the best potential for targeting. Bioinformatics analysis provides special insight into the pathobiology and the stage-specific expression of metastatic genes, but further elucidation is needed to take advantage of the metastatic cascade in a clinical setting. Future studies will likely incorporate these computational approaches to accomplish this in PC and other cancers.

## References

[1] Rawla P, Sunkara T, Gaduputi V (2019). Epidemiology of pancreatic cancer: Global Trends, etiology and risk factors. World Journal Oncology.

[2] Sung H, Ferlay J, Siegel RL, Laversanne M, Soerjomataram I, Jemal A (2021). Global cancer statistics 2020: GLOBOCAN estimates of incidence and mortality worldwide for 36 cancers in 185 countries. CA: A Cancer Journal for Clinicians.

[3] Siegel RL, Miller KD, Fuchs HE, Jemal A (2021). Cancer Statistics, 2021. CA: A Cancer Journal for Clinicians.

[4] Ilic M, Ilic I (2016). Epidemiology of pancreatic cancer. World Journal of Gastroenterology.

[5] Risch HA (2019). Diabetes and pancreatic cancer: Both cause and effect. Journal of the National Cancer Institute.

[6] Bailey P, Chang DK, Nones K, Johns AL, Patch AM, Gingras MC (2016). Genomic analyses identify molecular subtypes of pancreatic cancer. Nature.

[7] Moffitt RA, Marayati R, Flate EL, Volmar KE, Loeza SG, Hoadley KA (2015). Virtual microdissection identifies distinct tumor- and stroma-specific subtypes of pancreatic ductal adenocarcinoma. Nature Genetics.

[8] Collisson EA, Sadanandam A, Olson P, Gibb WJ, Truitt M, Gu S (2011). Subtypes of pancreatic ductal adenocarcinoma and their differing responses to therapy. Nature Medicine.

[9] Ryan DP, Hong TS, Bardeesy N (2014). Pancreatic adenocarcinoma. New England Journal of Medicine.

[10] Mizrahi JD, Surana R, Valle JW, Shroff RT (2020). Pancreatic cancer. Lancet.

[11] Hapach LA, Mosier JA, Wang W, Reinhart-King CA (2019). Engineered models to parse apart the metastatic cascade. NPJ Precision Oncology.

[12] Ganesh K, Massague J (2021). Targeting metastatic cancer. Nature Medicine.

[13] Hanahan D, Weinberg RA (2011). Hallmarks of cancer: The next generation. Cell.

[14] Maitra A (2019). Molecular envoys pave the way for pancreatic cancer to invade the liver. Nature.

[15] Massague J, Obenauf AC (2016). Metastatic colonization by circulating tumour cells. Nature.

[16] Lambert AW, Pattabiraman DR, Weinberg RA (2017). Emerging biological principles of metastasis. Cell.

[17] Obenauf AC, Massague J (2015). Surviving at a distance: Organ-specific metastasis. Trends in Cancer.

[18] Dai Z, Gu XY, Xiang SY, Gong DD, Man CF, Fan Y (2020). Research and application of single-cell sequencing in tumor heterogeneity and drug resistance of circulating tumor cells. Biomarker Research.

[19] Yang C, Xia BR, Jin WL, Lou G (2019). Circulating tumor cells in precision oncology: Clinical applications in liquid biopsy and 3D organoid model. Cancer Cell International.

[20] Jiang WG (1996). E-cadherin and its associated protein catenins, cancer invasion and metastasis. British Journal of Surgery.

[21] Klein CA (2009). Parallel progression of primary tumours and metastases. Nature Reviews Cancer.

[22] Caswell DR, Swanton C (2017). The role of tumour heterogeneity and clonal cooperativity in metastasis, immune evasion and clinical outcome. BMC Medicine.

[23] Turajlic S, Swanton C (2016). Metastasis as an evolutionary process. Science.

[24] Pelosi E, Castelli G, Testa U (2017). Pancreatic cancer: Molecular characterization, clonal evolution and cancer stem cells. Biomedicines.

[25] Bhandari V, Li CH, Bristow RG, Boutros PC, Consortium P (2020). Divergent mutational processes distinguish hypoxic and normoxic tumours.. Nature Communications.

[26] Muz B, de la Puente P, Azab F, Azab AK (2015). The role of hypoxia in cancer progression, angiogenesis, metastasis, and resistance to therapy. Hypoxia (Auckl).

[27] Tiwari A, Tashiro K, Dixit A, Soni A, Vogel K, Hall B (2020). Loss of HIF1A from pancreatic cancer cells increases expression of PPP1R1B and degradation of p53 to promote invasion and metastasis. Gastroenterology.

[28] Wang M, Liu J, Zhao Y, He R, Xu X, Guo X (2020). Upregulation of METTL14 mediates the elevation of PERP mRNA N(6) adenosine methylation promoting the growth and metastasis of pancreatic cancer. Molecular Cancer.

[29] Filipazzi P, Burdek M, Villa A, Rivoltini L, Huber V (2012). Recent advances on the role of tumor exosomes in immunosuppression and disease progression. Seminars in Cancer Biology.

[30] Sun W, Ren Y, Lu Z, Zhao X (2020). The potential roles of exosomes in pancreatic cancer initiation and metastasis. Molecular Cancer.

[31] Stoletov K, Beatty PH, Lewis JD (2020). Novel therapeutic targets for cancer metastasis. Expert Review of Anticancer Therapy.

[32] Chen MT, Sun HF, Zhao Y, Fu WY, Yang LP, Gao SP (2017). Comparison of patterns and prognosis among distant metastatic breast cancer patients by age groups: A SEER population-based analysis. Science and Reports.

[33] Leek RD, Lewis CE, Whitehouse R, Greenall M, Clarke J, Harris AL (1996). Association of macrophage infiltration with angiogenesis and prognosis in invasive breast carcinoma. Cancer Research.

[34] Wang H, Zhang C, Zhang J, Kong L, Zhu H, Yu J (2017). The prognosis analysis of different metastasis pattern in patients with different breast cancer subtypes: A SEER based study. Oncotarget.

[35] Smith MR, Mehra M, Nair S, Lawson J, Small EJ (2020). Relationship between metastasis-free survival and overall survival in patients with nonmetastatic castration-resistant prostate cancer. Clinical Genitourinary Cancer.

[36] Deng J, Chu X, Ren Z, Wang B (2020). Relationship between T stage and survival in distantly metastatic esophageal cancer: A STROBE-compliant study. Medicine (Baltimore).

[37] Yang J, Lu Z, Li L, Li Y, Tan Y, Zhang D (2020). Relationship of lymphovascular invasion with lymph node metastasis and prognosis in superficial esophageal carcinoma: Systematic review and meta-analysis. BMC Cancer.

[38] Zhan H, Zhao X, Lu Z, Yao Y, Zhang X (2021). Correlation and survival analysis of distant metastasis site and prognosis in patients with hepatocellular carcinoma. Frontiers in Oncology.

[39] Qian Y, Gong Y, Fan Z, Luo G, Huang Q, Deng S (2020). Molecular alterations and targeted therapy in pancreatic ductal adenocarcinoma. Journal of Hematology & Oncology.

[40] Beerenwinkel N, Greenman CD, Lagergren J (2016). Computational cancer biology: An evolutionary perspective. PLoS Computational Biology.

[41] Nagarajan N, Yapp EKY, Le NQK, Kamaraj B, Al-Subaie AM, Yeh HY (2019). Application of computational biology and artificial intelligence technologies in cancer precision drug discovery. BioMed Research International.

[42] Langfelder P, Horvath S (2008). WGCNA: An R package for weighted correlation network analysis. BMC Bioinformatics.

[43] Nguyen TM, Shafi A, Nguyen T, Draghici S (2019). Identifying significantly impacted pathways: A comprehensive review and assessment. Genome Biology.

[44] Luo L, Li Y, Huang C, Lin Y, Su Y, Cen H (2021). A new 7-gene survival score assay for pancreatic cancer patient prognosis prediction. American Journal of Cancer Research.

[45] Jin D, Jiao Y, Ji J, Jiang W, Ni W, Wu Y (2020). Identification of prognostic risk factors for pancreatic cancer using bioinformatics analysis. PeerJ.

[46] Xu JS, Liao KL, Wang X, He J, Wang XZ (2020). Combining bioinformatics techniques to explore the molecular mechanisms involved in pancreatic cancer metastasis and prognosis. Journal of Cellular and Molecular Medicine.

[47] Chen Q, Yu D, Zhao Y, Qiu J, Xie Y, Tao M (2019). Screening and identification of hub genes in pancreatic cancer by integrated bioinformatics analysis. Journal of Cellular Biochemistry.

[48] Wu J, Li Z, Zeng K, Wu K, Xu D, Zhou J (2019). Key genes associated with pancreatic cancer and their association with outcomes: A bioinformatics analysis. Molecular Medicine Reports.

[49] Zhou J, Hui X, Mao Y, Fan L (2019). Identification of novel genes associated with a poor prognosis in pancreatic ductal adenocarcinoma via a bioinformatics analysis. Bioscience Reports.

[50] Lu W, Li N, Liao F (2019). Identification of key genes and pathways in pancreatic cancer gene expression profile by integrative analysis. Genes (Basel).

[51] Wu M, Li X, Zhang T, Liu Z, Zhao Y (2019). Identification of a nine-gene signature and establishment of a prognostic nomogram predicting overall survival of pancreatic cancer. Frontiers in Oncology.

[52] Ma Y, Pu Y, Peng L, Luo X, Xu J, Peng Y (2019). Identification of potential hub genes associated with the pathogenesis and prognosis of pancreatic duct adenocarcinoma using bioinformatics meta-analysis of multi-platform datasets. Oncology Letters.

[53] Li Y, Zhu YY, Dai GP, Wu DJ, Gao ZZ, Zhang L (2019). Screening and validating the core biomarkers in patients with pancreatic ductal adenocarcinoma. Mathematical Biosciences and Engineering.

[54] Lu Y, Li C, Chen H, Zhong W (2018). Identification of hub genes and analysis of prognostic values in pancreatic ductal adenocarcinoma by integrated bioinformatics methods. Molecular Biology Reports.

[55] Xu J, Zheng H, Yuan S, Zhou B, Zhao W, Pan Y (2019). Overexpression of ANLN in lung adenocarcinoma is associated with metastasis. Thoracic Cancer.

[56] Wang A, Dai H, Gong Y, Zhang C, Shu J, Luo Y (2019). ANLN-induced EZH2 upregulation promotes pancreatic cancer progression by mediating miR-218-5p/LASP1 signaling axis. Journal of Experimental & Clinical Cancer Research.

[57] Janiszewska M, Primi MC, Izard T (2020). Cell adhesion in cancer: Beyond the migration of single cells. Journal of Biological Chemistry.

[58] Heerboth S, Housman G, Leary M, Longacre M, Byler S, Lapinska K (2015). EMT and tumor metastasis. Clinical and Translational Medicine.

[59] Idichi T, Seki N, Kurahara H, Yonemori K, Osako Y, Arai T (2017). Regulation of actin-binding protein ANLN by antitumor miR-217 inhibits cancer cell aggressiveness in pancreatic ductal adenocarcinoma. Oncotarget.

[60] Zeng S, Yu X, Ma C, Song R, Zhang Z, Zi X (2017). Transcriptome sequencing identifies ANLN as a promising prognostic biomarker in bladder urothelial carcinoma. Science and Reports.

[61] Guo HH, Wang YZ, Zhang ZK, Li MZ, Tian XD, Yang YM (2020). High mobility group AT-hook 2 promotes tumorigenicity of pancreatic cancer cells via upregulating ANLN. Experimental Cell Research.

[62] Nie Y, Zhao Z, Chen M, Ma F, Fan Y, Kang Y (2021). Anillin is a prognostic factor and is correlated with genovariation in pancreatic cancer based on databases analysis. Oncology Letters.

[63] Kou F, Sun H, Wu L, Li B, Zhang B, Wang X (2020). TOP2A promotes lung adenocarcinoma cells’ malignant progression and predicts poor prognosis in lung adenocarcinoma. Journal of Cancer.

[64] Pei YF, Yin XM, Liu XQ (2018). TOP2A induces malignant character of pancreatic cancer through activating beta-catenin signaling pathway. Biochimica et Biophysica Acta, Molecular Basis of Disease.

[65] Wang B, Shen Y, Zou Y, Qi Z, Huang G, Xia S (2020). TOP2A promotes cell migration, invasion and epithelial-mesenchymal transition in cervical cancer via activating the PI3K/AKT signaling. Cancer Management and Research.

[66] Zeng S, Liu A, Dai L, Yu X, Zhang Z, Xiong Q (2019). Prognostic value of TOP2A in bladder urothelial carcinoma and potential molecular mechanisms. BMC Cancer.

[67] Zhou Z, Liu S, Zhang M, Zhou R, Liu J, Chang Y (2017). Overexpression of topoisomerase 2-alpha confers a poor prognosis in pancreatic adenocarcinoma identified by co-expression analysis. Digestive Diseases and Sciences.

[68] Banyard J, Chung I, Migliozzi M, Phan DT, Wilson AM, Zetter BR (2014). Identification of genes regulating migration and invasion using a new model of metastatic prostate cancer. BMC Cancer.

[69] Jo M, Lester RD, Montel V, Eastman B, Takimoto S, Gonias SL (2009). Reversibility of epithelial-mesenchymal transition (EMT) induced in breast cancer cells by activation of urokinase receptor-dependent cell signaling. Journal of Biological Chemistry.

[70] Nguyen DH, Hussaini IM, Gonias SL (1998). Binding of urokinase-type plasminogen activator to its receptor in MCF-7 cells activates extracellular signal-regulated kinase 1 and 2 which is required for increased cellular motility. Journal of Biological Chemistry.

[71] Webb DJ, Nguyen DH, Gonias SL (2000). Extracellular signal-regulated kinase functions in the urokinase receptor-dependent pathway by which neutralization of low density lipoprotein receptor-related protein promotes fibrosarcoma cell migration and matrigel invasion. Journal of Cell Science.

[72] Liu P, Weng Y, Sui Z, Wu Y, Meng X, Wu M (2016). Quantitative secretomic analysis of pancreatic cancer cells in serum-containing conditioned medium. Science and Reports.

[73] Xue A, Xue M, Jackson C, Smith RC (2009). Suppression of urokinase plasminogen activator receptor inhibits proliferation and migration of pancreatic adenocarcinoma cells via regulation of ERK/p38 signaling. International Journal of Biochemistry & Cell Biology.

[74] Wang W, Abbruzzese JL, Evans DB, Chiao PJ (1999). Overexpression of urokinase-type plasminogen activator in pancreatic adenocarcinoma is regulated by constitutively activated RelA. Oncogene.

[75] Skandalis SS, Kletsas D, Kyriakopoulou D, Stavropoulos M, Theocharis DA (2006). The greatly increased amounts of accumulated versican and decorin with specific post-translational modifications may be closely associated with the malignant phenotype of pancreatic cancer. Biochimica et Biophysica Acta.

[76] Sakko AJ, Ricciardelli C, Mayne K, Suwiwat S, LeBaron RG, Marshall VR (2003). Modulation of prostate cancer cell attachment to matrix by versican. Cancer Research.

[77] Ricciardelli C, Brooks JH, Suwiwat S, Sakko AJ, Mayne K, Raymond WA (2002). Regulation of stromal versican expression by breast cancer cells and importance to relapse-free survival in patients with node-negative primary breast cancer. Clinical Cancer Research.

[78] Touab M, Villena J, Barranco C, Arumi-Uria M, Bassols A (2002). Versican is differentially expressed in human melanoma and may play a role in tumor development. American Journal of Pathology.

[79] Mitsui Y, Shiina H, Kato T, Maekawa S, Hashimoto Y, Shiina M (2017). Versican promotes tumor progression, metastasis and predicts poor prognosis in renal carcinoma. Molecular Cancer Research.

[80] Chida S, Okayama H, Noda M, Saito K, Nakajima T, Aoto K (2016). Stromal VCAN expression as a potential prognostic biomarker for disease recurrence in stage II-III colon cancer. Carcinogenesis.

[81] Brady JJ, Chuang CH, Greenside PG, Rogers ZN, Murray CW, Caswell DR (2016). An Arntl2-driven secretome enables lung adenocarcinoma metastatic self-sufficiency. Cancer Cell.

[82] Wang Z, Liu T, Xue W, Fang Y, Chen X, Xu L (2020). ARNTL2 promotes pancreatic ductal adenocarcinoma progression through TGF/BETA pathway and is regulated by miR-26a-5p. Cell Death & Disease.

[83] Lu M, Huang L, Tang Y, Sun T, Li J, Xiao S (2020). ARNTL2 knockdown suppressed the invasion and migration of colon carcinoma: Decreased SMOC2-EMT expression through inactivation of PI3K/AKT pathway. American Journal of Translational Research.

[84] Mazzoccoli G, Pazienza V, Panza A, Valvano MR, Benegiamo G, Vinciguerra M (2012). ARNTL2 and SERPINE1: Potential biomarkers for tumor aggressiveness in colorectal cancer. Journal of Cancer Research and Clinical Oncology.

[85] Xie F, Ling L, van Dam H, Zhou F, Zhang L (2018). TGF-beta signaling in cancer metastasis. Acta Biochimica et Biophysica Sinica (Shanghai).

[86] Gkretsi V, Stylianopoulos T (2018). Cell adhesion and matrix stiffness: Coordinating cancer cell invasion and metastasis. Frontiers in Oncology.

[87] Maziveyi M, Alahari SK (2017). Cell matrix adhesions in cancer: The proteins that form the glue. Oncotarget.

[88] An X, Xu F, Luo R, Zheng Q, Lu J, Yang Y (2018). The prognostic significance of topoisomerase II alpha protein in early stage luminal breast cancer. BMC Cancer.

[89] Li X, Liu Y, Chen W, Fang Y, Xu H, Zhu HH (2014). TOP2Ahigh is the phenotype of recurrence and metastasis whereas TOP2Aneg cells represent cancer stem cells in prostate cancer. Oncotarget.

[90] Brase JC, Schmidt M, Fischbach T, Sultmann H, Bojar H, Koelbl H (2010). ERBB2 and TOP2A in breast cancer: A comprehensive analysis of gene amplification, RNA levels, and protein expression and their influence on prognosis and prediction. Clinical Cancer Research.

[91] Kirk JS, Schaarschuch K, Dalimov Z, Lasorsa E, Ku S, Ramakrishnan S (2015). Top2a identifies and provides epigenetic rationale for novel combination therapeutic strategies for aggressive prostate cancer. Oncotarget.

[92] Frandsen TL, Holst-Hansen C, Nielsen BS, Christensen IJ, Nyengaard JR, Carmeliet P (2001). Direct evidence of the importance of stromal urokinase plasminogen activator (uPA) in the growth of an experimental human breast cancer using a combined uPA gene-disrupted and immunodeficient xenograft model. Cancer Research.

[93] Wang X, Jiang Z, An J, Mao X, Lin F, Sun P (2018). Effect of a synthetic inhibitor of urokinase plasminogen activator on the migration and invasion of human cervical cancer cells *in vitro*. Molecular Medicine Reports.

[94] Lemmon MA, Schlessinger J (2010). Cell signaling by receptor tyrosine kinases. Cell.

[95] Hudis CA (2007). Trastuzumab-Mechanism of action and use in clinical practice. New England Journal of Medicine.

[96] Tripathy D, Slamon DJ, Cobleigh M, Arnold A, Saleh M, Mortimer JE (2004). Safety of treatment of metastatic breast cancer with trastuzumab beyond disease progression. Journal of Clinical Oncology.

[97] Montemurro F, Donadio M, Clavarezza M, Redana S, Jacomuzzi ME, Valabrega G (2006). Outcome of patients with HER2-positive advanced breast cancer progressing during trastuzumab-based therapy. The Oncologist.

[98] Ardito CM, Gruner BM, Takeuchi KK, Lubeseder-Martellato C, Teichmann N, Mazur PK (2012). EGF receptor is required for KRAS-induced pancreatic tumorigenesis. Cancer Cell.

[99] Kaushik G, Seshacharyulu P, Rauth S, Nallasamy P, Rachagani S, Nimmakayala RK (2021). Selective inhibition of stemness through EGFR/FOXA2/SOX9 axis reduces pancreatic cancer metastasis. Oncogene.

[100] Skoulidis F, Papadimitrakopoulou VA (2017). Targeting the gatekeeper: Osimertinib in EGFR T790M mutation-positive non-small cell lung cancer. Clinical Cancer Research.

[101] Wu P, Nielsen TE, Clausen MH (2015). FDA-approved small-molecule kinase inhibitors. Trends in Pharmacological Sciences.

[102] Agustoni F, Suda K, Yu H, Ren S, Rivard CJ, Ellison K (2019). EGFR-directed monoclonal antibodies in combination with chemotherapy for treatment of non-small-cell lung cancer: An updated review of clinical trials and new perspectives in biomarkers analysis. Cancer Treatment Reviews.

[103] Russo A, Franchina T, Ricciardi GR, Picone A, Ferraro G, Zanghi M (2015). A decade of EGFR inhibition in EGFR-mutated non small cell lung cancer (NSCLC): Old successes and future perspectives. Oncotarget.

[104] Wu P, Clausen MH, Nielsen TE (2015). Allosteric small-molecule kinase inhibitors. Pharmacology & Therapeutics.

[105] Thomas SK, Lee J, Beatty GL (2020). Paracrine and cell autonomous signalling in pancreatic cancer progression and metastasis. eBioMedicine.

[106] Christofori G (2006). New signals from the invasive front. Nature.

[107] Cavallaro U, Christofori G (2004). Cell adhesion and signalling by cadherins and Ig-CAMs in cancer. Nature Reviews Cancer.

[108] Kalluri R, Weinberg RA (2009). The basics of epithelial-mesenchymal transition. The Journal of Clinical Investigation.

[109] Rodriguez-Aznar E, Wiesmuller L, Sainz B, Hermann PC (2019). EMT and stemness-Key players in pancreatic cancer stem cells. Cancers (Basel).

[110] Forte E, Chimenti I, Rosa P, Angelini F, Pagano F, Calogero A (2017). EMT/MET at the crossroad of stemness, regeneration and oncogenesis: The ying-yang equilibrium recapitulated in cell spheroids. Cancers (Basel).

[111] Procacci P, Moscheni C, Sartori P, Sommariva M, Gagliano N (2018). Tumor(-)stroma cross-talk in human pancreatic ductal adenocarcinoma: A focus on the effect of the extracellular matrix on tumor cell phenotype and invasive potential. Cells.

[112] Fucikova J, Kasikova L, Truxova I, Laco J, Skapa P, Ryska A (2018). Relevance of the chaperone-like protein calreticulin for the biological behavior and clinical outcome of cancer. Immunology Letters.

[113] Sheng W, Wang G, Tang J, Shi X, Cao R, Sun J (2020). Calreticulin promotes EMT in pancreatic cancer via mediating Ca(2+) dependent acute and chronic endoplasmic reticulum stress. Journal of Experimental & Clinical Cancer Research.

[114] Sheng W, Chen C, Dong M, Wang G, Zhou J, Song H (2017). Calreticulin promotes EGF-induced EMT in pancreatic cancer cells via Integrin/EGFR-ERK/MAPK signaling pathway. Cell Death & Disease.

[115] Begum A, Ewachiw T, Jung C, Huang A, Norberg KJ, Marchionni L (2017). The extracellular matrix and focal adhesion kinase signaling regulate cancer stem cell function in pancreatic ductal adenocarcinoma. PLoS ONE.

[116] Pitarresi JR, Rustgi AK (2019). Mechanisms underlying metastatic pancreatic cancer. Advances in Experimental Medicine and Biology.

[117] Lawlor RT, Veronese N, Nottegar A, Malleo G, Smith L, Demurtas J (2019). Prognostic role of high-grade tumor budding in pancreatic ductal adenocarcinoma: A systematic review and meta-analysis with a focus on epithelial to mesenchymal transition. Cancers (Basel).

[118] Hynes RO (2002). Integrins: Bidirectional, allosteric signaling machines. Cell.

[119] Miranti CK, Brugge JS (2002). Sensing the environment: A historical perspective on integrin signal transduction. Nature Cell Biology.

[120] Giancotti FG, Ruoslahti E (1999). Integrin signaling. Science.

[121] Hynes RO (1992). Integrins: Versatility, modulation, and signaling in cell adhesion. Cell.

[122] Grzesiak JJ, Ho JC, Moossa AR, Bouvet M (2007). The integrin-extracellular matrix axis in pancreatic cancer. Pancreas.

[123] Sawai H, Okada Y, Funahashi H, Matsuo Y, Takahashi H, Takeyama H (2005). Activation of focal adhesion kinase enhances the adhesion and invasion of pancreatic cancer cells via extracellular signal-regulated kinase-1/2 signaling pathway activation. Molecular Cancer.

[124] Brannon A, Drouillard D, Steele N, Schoettle S, Abel EV, Crawford HC (2020). Beta 1 integrin signaling mediates pancreatic ductal adenocarcinoma resistance to MEK inhibition. Science and Reports.

[125] Jin S, Lee WC, Aust D, Pilarsky C, Cordes N (2019). beta8 integrin mediates pancreatic cancer cell radiochemoresistance. Molecular Cancer Research.

[126] Schlieman MG, Fahy BN, Ramsamooj R, Beckett L, Bold RJ (2003). Incidence, mechanism and prognostic value of activated AKT in pancreas cancer. British Journal of Cancer.

[127] Asano T, Yao Y, Zhu J, Li D, Abbruzzese JL, Reddy SA (2004). The PI 3-kinase/Akt signaling pathway is activated due to aberrant Pten expression and targets transcription factors NF-kappaB and c-Myc in pancreatic cancer cells. Oncogene.

[128] Ruggeri BA, Huang L, Wood M, Cheng JQ, Testa JR (1998). Amplification and overexpression of the AKT2 oncogene in a subset of human pancreatic ductal adenocarcinomas. Molecular Carcinogenesis.

[129] Cheng JQ, Ruggeri B, Klein WM, Sonoda G, Altomare DA, Watson DK (1996). Amplification of AKT2 in human pancreatic cells and inhibition of AKT2 expression and tumorigenicity by antisense RNA. Proceedings of the National Academy of Sciences.

[130] Willems L, Tamburini J, Chapuis N, Lacombe C, Mayeux P, Bouscary D (2012). PI3K and mTOR signaling pathways in cancer: New data on targeted therapies. Current Oncology Reports.

[131] Duluc C, Moatassim-Billah S, Chalabi-Dchar M, Perraud A, Samain R, Breibach F (2015). Pharmacological targeting of the protein synthesis mTOR/4E-BP1 pathway in cancer-associated fibroblasts abrogates pancreatic tumour chemoresistance. EMBO Mol Med.

[132] Graupera M, Guillermet-Guibert J, Foukas LC, Phng LK, Cain RJ, Salpekar A (2008). Angiogenesis selectively requires the p110alpha isoform of PI3K to control endothelial cell migration. Nature.

[133] Kaneda MM, Cappello P, Nguyen AV, Ralainirina N, Hardamon CR, Foubert P (2016). Macrophage PI3Kgamma drives pancreatic ductal adenocarcinoma progression. Cancer Discovery.

[134] Jiang N, Dai Q, Su X, Fu J, Feng X, Peng J (2020). Role of PI3K/AKT pathway in cancer: The framework of malignant behavior. Molecular Biology Reports.

[135] Murthy D, Attri KS, Singh PK (2018). Phosphoinositide 3-kinase signaling pathway in pancreatic ductal adenocarcinoma progression, pathogenesis, and therapeutics. Frontiers in Physiology.

[136] Hanahan D, Weinberg RA (2000). The hallmarks of cancer. Cell.

[137] Fouad YA, Aanei C (2017). Revisiting the hallmarks of cancer. American Journal of Cancer Research.

[138] Mukherjee R, Vanaja KG, Boyer JA, Gadal S, Solomon H, Chandarlapaty S (2021). Regulation of PTEN translation by PI3K signaling maintains pathway homeostasis. Molecular Cell.

[139] Liu P, Cheng H, Roberts TM, Zhao JJ (2009). Targeting the phosphoinositide 3-kinase pathway in cancer. Nature Reviews. Drug Discovery.

[140] Hollander MC, Blumenthal GM, Dennis PA (2011). PTEN loss in the continuum of common cancers, rare syndromes and mouse models. Nature Reviews Cancer.

[141] Babiker HM, Karass M, Recio-Boiles A, Chandana SR, McBride A, Mahadevan D (2019). Everolimus for the treatment of advanced pancreatic ductal adenocarcinoma (PDAC). Expert Opinion on Investigational Drugs.

[142] Khatri P, Draghici S (2005). Ontological analysis of gene expression data: Current tools, limitations, and open problems. Bioinformatics.

